# Attention driven deep convolutional network with optimized learning for accurate landslide detection and monitoring

**DOI:** 10.1038/s41598-026-36737-2

**Published:** 2026-01-30

**Authors:** Sangeetha S.K.B, Krishnammal N, Pavan Kumar M R, Sandeep Kumar Mathivanan, Shakila Basheer, Amira Elsir Tayfour Ahmed

**Affiliations:** 1 Manipal Institute of Technology Bengaluru, Manipal Academy of Higher Education, Manipal, India; 2 Department of Computer Science and Engineering, Vel Tech Rangarajan Dr. Sagunthala R&D Institute of Science and Technology, Avadi, Chennai, Tamil Nadu India; 3 Department of Computer Science and Engineering, Sree Rama Engineering College, Tirupati, India; 4 School of Computing Science and Engineering, Galgotias University, Greater Noida, 203201 India; 5 Department of Information Systems, College of Computer and Information Science, Princess Nourah bint Abdulrahman University, P.O. BOX 84428, Riyadh, 11671 Saudi Arabia; 6 Technical and Engineering Specialities Unit, Applied College, King Khalid University, Mohyel Asser, Saudi Arabia

**Keywords:** Deep neural network, ImageNet, Landslide monitoring, NASA landslide dataset, VGG16, Environmental sciences, Environmental impact

## Abstract

Effective landslide monitoring is essential for mitigating risks to infrastructure and communities, particularly in geologically unstable regions. Traditional monitoring methods, such as ground surveys and visual inspections, are time-intensive and lack early detection capabilities. To address these limitations, this study employs feature fusion and enhanced Deep Convolutional Neural Networks (DCNNs) for landslide detection. The model is built upon a fine-tuned, pre-trained VGG16 architecture, adapted to a new landslide dataset. Key modifications include the integration of a spatial attention mechanism, optimized learning rate schedules, attention-based Global Average Pooling (GAP), and the Lookahead Adam optimizer, all aimed at improving feature extraction, model convergence, and generalization. Experimental results demonstrate that the proposed approach achieves high accuracy, with performance ranging from 90% to 96% across different datasets and training iterations. Using the Kaggle Landslide Dataset, the model attained a training accuracy of 93%, with validation and testing accuracies of 95.2% and 95.8%, respectively. Comparable results were observed with the NASA Landslide Inventory, confirming the robustness of the method. The findings highlight the potential of DCNN-based models, augmented with attention mechanisms, as a reliable and efficient tool for landslide monitoring, significantly outperforming conventional assessment methods.

## Introduction

Landslides are one of the most critical geohazards that cause massive infrastructure loss and fatalities^1^. The devastating effect of such disasters is clear from recent catastrophes like the failure of the Vajont Dam in Italy in 1963, when an enormous landslide produced a devastating wave, and the 2015 Gorkha earthquake in Nepal, which led to multiple secondary landslides^2,3^. The necessity for effective monitoring and early warning systems to reduce risks was further highlighted by the widespread loss of property and loss of lives caused by monsoon-induced landslides in Kerala, India, in 2018 and 2019^4–6^.

Ground surveys, visual observation, and seismic monitoring are some of the traditional landslide monitoring techniques that have been used extensively^7^. While ground surveys permit comprehensive on-site investigations, they are laborious and unsuitable for repeated or extensive monitoring^8^. While visual observations permit surface real-time observations, they cannot be used to identify early-stage instabilities before large-scale failures have occurred^9,10^. Seismic monitoring is a well-established technique for obtaining critical data on subsurface acceleration, motion, displacement, and velocity associated with landslide processes. While high-density seismic arrays in the Himalayas enhance monitoring capabilities, spatial coverage gaps persist in sparsely populated and lightly instrumented regions, limiting observation capabilities in these areas^11–13^.

Recent advancements in Artificial Intelligence (AI) and remote sensing have enabled the creation of scalable and effective landslide detection methods to bridge these gaps^14^. Slight deformations in the ground can be detected using high-resolution spaceborne images that provide large-scale, contiguous measurements of ground changes^15^. Satellite monitoring coupled with Deep Convolutional Neural Networks (DCNNs) greatly enhances the accuracy of landslide predictions^16,17^. In image processing applications like feature extraction and classification, DCNNs have excelled. These models can accurately identify patterns that are indicative of slope instability by being trained on vast annotated datasets^18^.

By combining a VGG16-based DCNN model with high-resolution satellite images, this study provides an AI-driven method for landslide detection. To make the model more flexible to varied terrain, it is fine-tuned with specific datasets such as the NASA Landslide Inventory and the Kaggle Landslide Dataset. The proposed approach is better than conventional methods in terms of accuracy, efficiency, and real-time monitoring capability, as confirmed by experimental data. In spite of restrictions like overfitting and heterogeneity in the dataset, the results indicate that AI-based techniques provide a robust and scalable approach to risk prediction of landslides.

Due to its strong performance on medium-sized datasets, computational efficiency, and reduced architecture, VGG16 was chosen for this study. It does have a tendency to overfit if not correctly tuned. For a number of reasons, a number of models were considered but ultimately discarded. ResNet is less useful owing to its higher processing expenses and longer training times, even though it can train deeper networks and avoid vanishing gradients. Although DenseNet offers high accuracy and efficient feature reuse, its complexity makes it more susceptible to overfitting. While transformer-based models are very good at picking up on spatial correlations, their lengthy inference times and requirement for big labeled datasets render them unsuitable for the scope of this research. Ultimately, a modified VGG16 architecture was chosen as the base due to its balance of simplicity, accuracy, and computational efficiency. To improve its performance for landslide detection, we incorporated a spatial attention mechanism, optimized dense layers, and adaptive learning strategies, building a robust VGG16-derived model that maintains deployment feasibility while significantly enhancing generalization and accuracy.

Hybrid and ensemble methods have been explored in various studies on landslide detection^19–21^. Although a CNN + LSTM model is existing, the use of LSTM is not optimal since it is more suited to sequential data than to spatial image classification^22,23^. CNNs combined with Decision Tree Ensembles improved interpretability but introduced complexity. Decision Tree Ensembles were combined which improved interpretability but introduced complexity that hindered scaling the method^24,25^. A CNN + Transformer model needs computational requirements and the necessity of intensive pre-training^26,27^. In view of these aspects, the proposed system prioritizes a hybrid deep learning approach combining CNN, attention mechanisms, and dynamic learning rates. This supports improved generalization, reduced overfitting, and enhanced feature extraction without sacrificing computing efficiency.

Gomroki et al.^28^ presents STCD-EffV2T Unet, a semi-transfer learning approach using EfficientNetV2 T-Unet for urban and land cover change detection with Sentinel-2 satellite images. Gomroki et al.^29^ introduces UNet-GCViT, a UNet-based framework incorporating global context vision transformer blocks for building damage detection, emphasizing the effectiveness of transformer models in spatial context understanding. Gomroki et al.^30^ combines EfficientViTB and Yolov8 in the EMYNet-BDD architecture, designed for building damage detection using post-event remote sensing images, highlighting the synergy between vision transformers and YOLO for efficient encoding and decoding processes. These studies contribute to advancing remote sensing applications by leveraging innovative deep learning models for improved accuracy and efficiency in environmental monitoring tasks.

Many deep learning models, especially Convolutional Neural Networks (CNNs), have been investigated for landslide detection in recent years. Despite their demonstrated efficacy in image classification tasks, CNNs such as VGG16 are prone to overfitting, particularly when trained on small or unbalanced datasets. This restriction frequently leads to subpar generalization to novel or varied terrains. Furthermore, while ResNet and DenseNet models have better feature extraction capabilities, they are less appropriate for real-time deployment in resource-constrained applications because of their high computational overhead and lengthy training periods.

The suggested model expands on the VGG16 architecture, which balances computing performance and simplicity, to overcome these issues. We improve VGG16 by adding attention-based Global Average Pooling (GAP), optimal learning rate schedules, and a spatial attention mechanism. Without adding the high computational costs of deeper networks, these changes greatly increase feature extraction, reduce overfitting, and improve model generalization. With these improvements, the suggested model gets around the drawbacks of earlier studies and becomes a more effective landslide monitoring tool.

The study promotes proactive risk management and early warning through the development of an automated landslide detection system through the use of satellite imagery and sophisticated deep learning techniques. The research identifies how the integration of AI and remote sensing has the potential to enhance disaster preparedness and mitigate the dangers of landslides globally. This study proposes a VGG16-based model that is fine-tuned with spatial attention mechanisms and optimized learning configurations. Additionally, baseline CNN architectures with varying convolutional layers and filters were developed solely for comparative analysis.

The proposed work provides several contributions to the field of deep learning-based landslide detection. First, the VGG16 architecture’s incorporation of a spatial attention mechanism improves the model’s capacity to concentrate on important spatial areas in intricate terrains, hence increasing classification accuracy. Secondly, a multimodal feature fusion approach is used to create more discriminative feature space that captures a variety of terrain properties by merging pixel intensity histograms, GLCM-based texture features, and NDVI-based spectral indices. Third, by striking a balance between optimization stability and exploratory updates, the Lookahead Adam optimizer with dynamic learning rate schedules improves generalization performance while speeding up training convergence.

The main objectives of the study are.

1. To design an AI-based landslide detection framework that integrates high-resolution satellite imagery with a fine-tuned VGG16-based Deep Convolutional Neural Network (DCNN) for improved accuracy in identifying and monitoring landslide-prone areas.
2. To enhance detection precision and computational efficiency by incorporating a spatial attention mechanism and an optimized learning rate schedule, thereby achieving consistent performance ranging from 90% to 96% across multiple datasets.
3. To validate the robustness and generalizability of the proposed model through comprehensive evaluation on diverse datasets, including the Kaggle Landslide Dataset and NASA Landslide Inventory, ensuring applicability in varied geological terrains and real-world conditions.

## System methodology

The proposed system methodology for landslide detection comprises three essential modules: data acquisition and preprocessing, model development and fine-tuning, and evaluation. In the data acquisition and preprocessing module, high-resolution satellite imagery is collected, and datasets such as the Kaggle landslide dataset (https://www.kaggle.com/datasets/rajumavinmar/landslide-dataset) and the NASA landslide inventory (https://www.kaggle.com/datasets/kazushiadachi/global-landslide-data) are curated. This module includes critical preprocessing steps, such as data cleaning, normalization, to enhance the dataset’s quality for training DCNNs. The model development and fine-tuning module employs the VGG16 architecture, initially pre-trained on the ImageNet dataset, which is then fine-tuned with the preprocessed landslide datasets.

In the proposed system, the Spatial Attention Mechanism improves feature extraction by emphasizing appropriate spatial locations and enables the model to focus on important locations in landslide images. Batch Normalization improves training stability and convergence rates by normalizing activations and mitigating internal covariate shifts. The Lookahead Adam Optimizer is used to further optimize; it provides faster convergence and better generalization than the standard Adam optimizer. Besides, Attention-based Global Average Pooling (GAP) ensures that key convolutional features are preserved, enhancing the model’s ability for identifying important patterns for successful landslide detection.

Fine-tuning includes optimization of parameters like learning rate and batch size, while incorporating techniques like dropout and regularization to mitigate overfitting and improve model robustness. The evaluation and validation module focuses on assessing the performance of the fine-tuned DCNN model using metrics such as accuracy, precision, and recall. This involves a structured split of the data into training, validation, and test sets. The module also addresses overfitting concerns and employs methods like cross-validation and hyperparameter tuning to further refine the model’s performance.

### Data acquisition and preprocessing

**Fig. 1:**
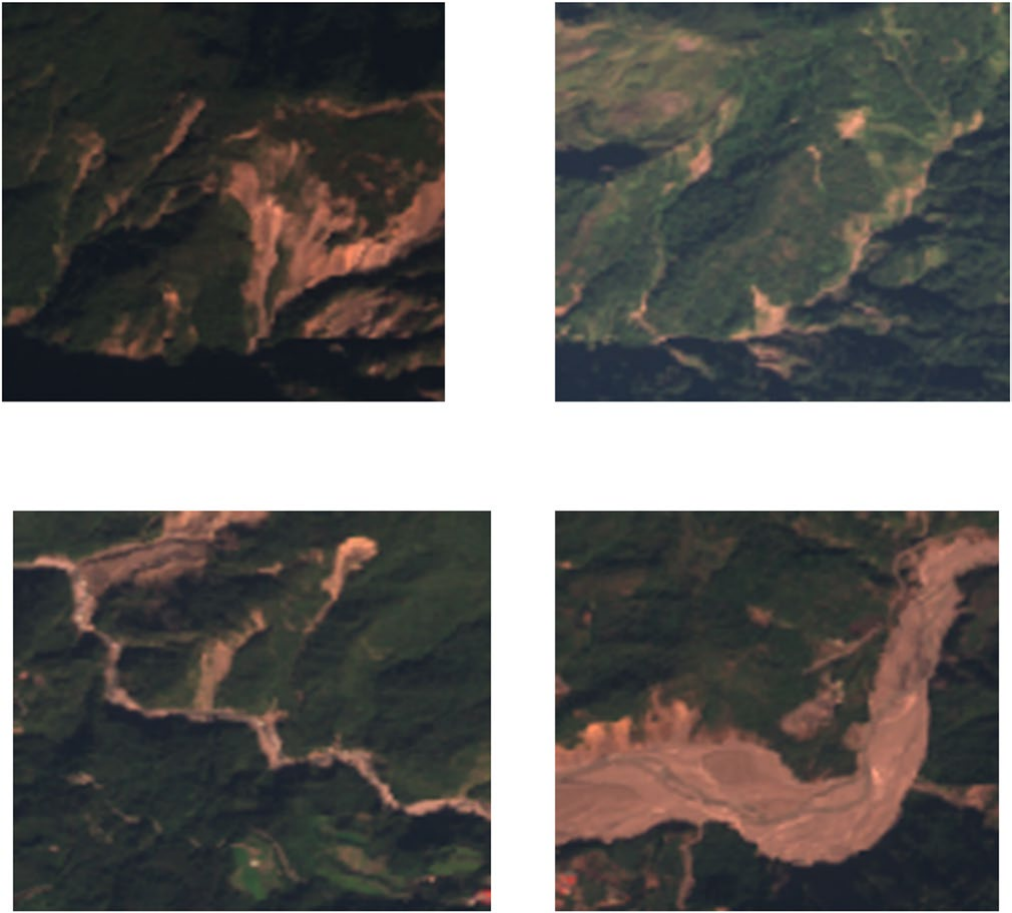
Sample images.

The Kaggle Landslide Dataset and the NASA Landslide Inventory are two geographically distinct datasets used in this study, each of which has special benefits for reliable model development and assessment. The Kaggle Landslide Dataset features 1,000 high-resolution (256 × 256 pixel) satellite images obtained from publicly available platforms such as Google Earth and Sentinel-2. The images cover a variety of terrains, including urban, rural, and mountainous areas in South America, Nepal, Indonesia, and India. The dataset is appropriate for balanced training since it contains 500 landslide and non-landslide samples, each with binary labels (0 for non-landslide, 1 for landslide). Sample image is shown in Fig. 1.

More than 1,500 geo-referenced and annotated satellite images with a pixel resolution of up to 512 × 512 are available in the NASA Landslide Inventory. The MODIS (250–500 m) and Landsat 8 (30 m) satellites are the sources of these images, which span Eastern Africa, the Pacific Rim (including portions of Japan and the Philippines), Central America, and South Asia (India, Nepal). It is a rich resource for spatiotemporal analysis because each entry contains metadata like the timestamp, location (latitude/longitude), trigger type (e.g., seismic, rainfall), and event severity.

**Fig. 2:**
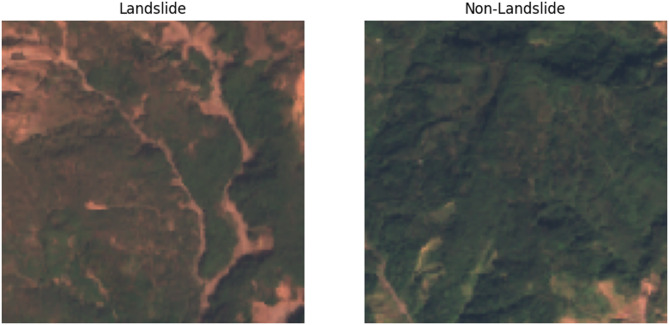
Landslide vs. non-landslide.

The objective of developing a generalizable and scalable landslide detection model that can function across various geological formations, climatic zones, and land-use patterns justifies the use of multi-regional datasets. By preventing the model from being overfit to a single area and enabling worldwide deployment across a variety of terrain, this method improves the model’s practicality in remote areas. Figure 2 depicts Landslide Vs Non-Landslide images.

#### Pixel intensity histogram analysis

Pixel intensity histograms were calculated for each data set to quantify the changes in topographic characteristics. According to the analysis, landslide-damaged regions have clearly defined peaks in intensity distributions corresponding to changes in the landscape, such as erosion, sediment transport, or vegetation loss. Histograms in non-landslide areas typically represent a single, well-defined peak and are more regular, indicating stable ground. Mean intensity, standard deviation, entropy, skewness, kurtosis, and zero crossings are some of the important statistical parameters restored (Table 1). In particular, high skewness or kurtosis due to sudden terrain variations and complex terrain variations often related to landslides are represented by higher values of entropy.

#### Texture feature extraction using GLCM

Gray Level Co-occurrence Matrix (GLCM) analysis was employed to capture textural variations in an attempt to enhance classification accuracy even further. The GLCM extracts four key features: Contrast brings to the fore abrupt changes in the terrain; higher values indicate areas that have experienced landslides. Lower correlation values represent uneven terrain patterns characteristic of landslides. Correlation captures textural homogeneity. Texture homogeneity is represented by energy; lower energy values signify rough, disordered surfaces. In homogeneity, lower values indicate rugged, landslide-promoting regions, whereas higher values signify flat ground. In comparison to stable zones, landslide images generally display stronger contrast, weaker correlation, and smaller homogeneity, evidenced by the values in Table 2 derived from the GLCM. These findings are corroborated by the histogram analysis, which indicates more complicated texture and intensity patterns for landslide-prone areas.

**Table 1:** Example values for histograms.

Feature	Image 1	Image 2	Image 3	Image 4	Image 5	Image 6	Image 7	Image 8	Image 9
Mean Intensity	50	128	70	200	100	150	90	180	120
Standard Deviation	20	30	25	35	20	40	30	30	25
Histogram Entropy	4.0	4.5	3.8	4.2	4.1	4.3	4.0	4.4	4.1
Peak Count	2	1	2	3	2	1	2	2	2
Peak Intensity	50, 200	128	70, 180	200, 50	100, 150	150	90, 150	180, 60	120, 180
Skewness	0.1	0.2	0.0	-0.1	0.2	0.3	-0.2	0.1	0.0
Kurtosis	2.8	3.0	2.5	3.2	2.9	3.1	2.7	3.0	2.8
Contrast	150	100	120	150	130	160	140	120	140
Dynamic Range	180	130	140	170	150	180	160	140	150
Zero Crossings	2	1	3	4	2	2	3	3	2

**Table 2:** Example values - GLCM Analysis.

Image ID	Contrast	Correlation	Energy	Homogeneity
1	12.34	0.85	0.78	0.56
2	15.67	0.78	0.65	0.50
3	8.91	0.82	0.80	0.62
4	20.23	0.74	0.55	0.47
5	14.56	0.79	0.70	0.53
6	22.34	0.71	0.60	0.45
7	9.87	0.83	0.77	0.60
8	18.12	0.76	0.68	0.48
9	13.45	0.80	0.72	0.51

**Fig. 3:**
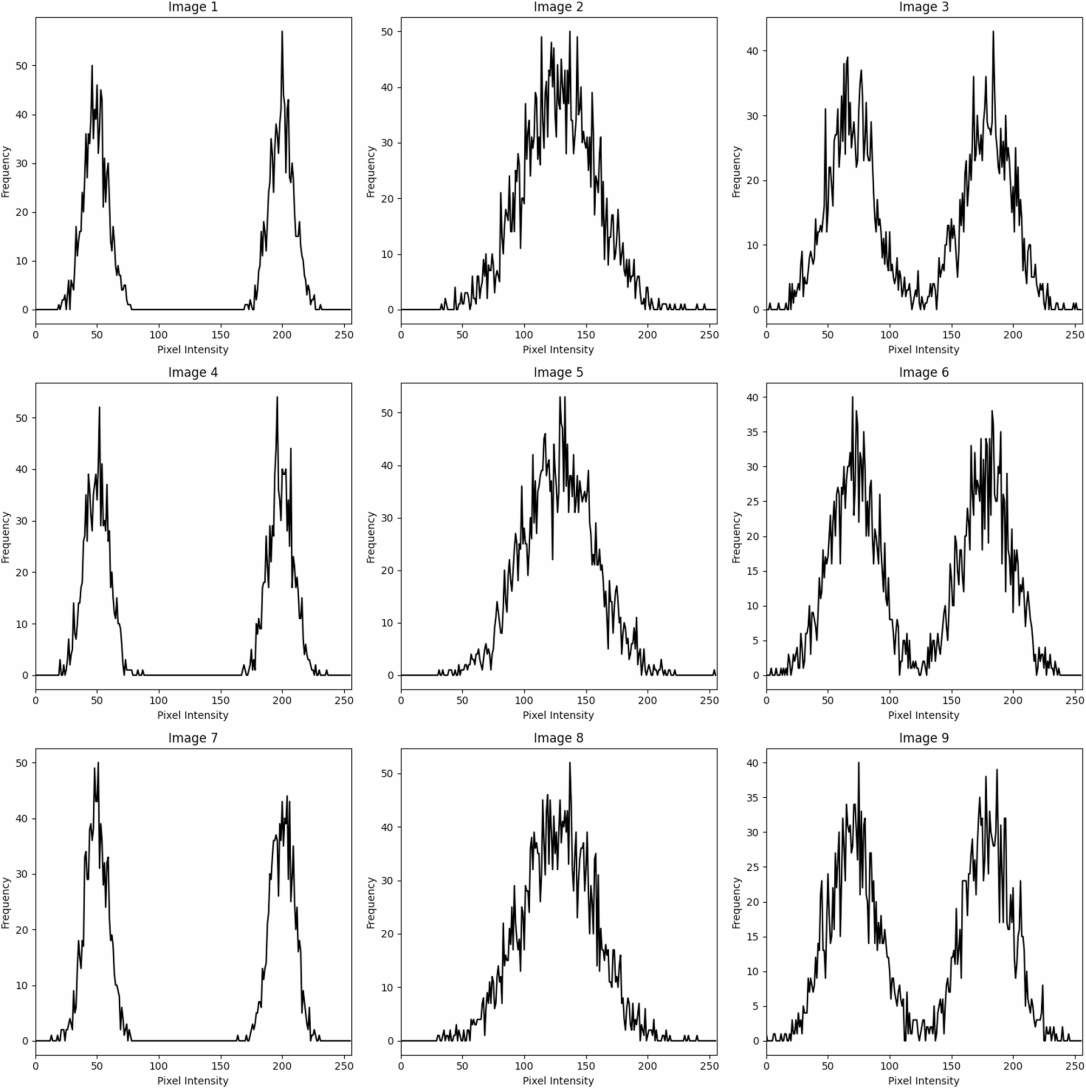
Pixel intensities - Sample.

**Fig. 4:**
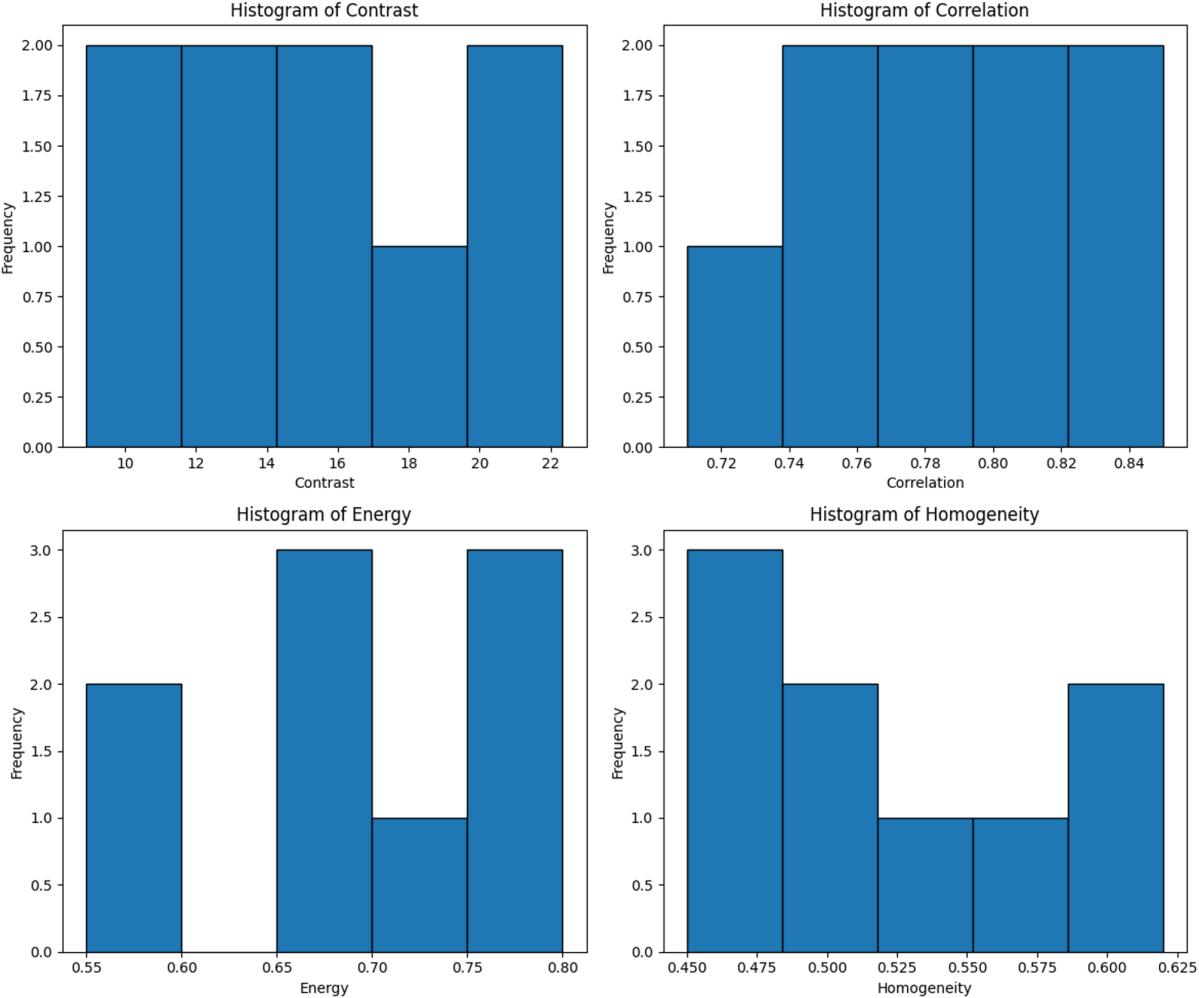
Distribution of texture feature across images.

In order to preserve consistency and lower the computational cost during training, all satellite images were shrunk to 256 × 256 pixels. To guarantee consistency in the model input, pixel values in each image were scaled to the interval [0, 1]. The training set was also subjected to data augmentation methods like brightness tweaks, random rotations, and horizontal flipping in order to broaden the dataset’s diversity and enhance model generalization. A VGG16-based deep learning model was built utilizing the extracted histogram and GLCM features. Whereas GLCM provided spatial texture context and improved classification performance, the histogram analysis revealed global intensity distribution patterns. The performance of landslide identification was enhanced through the integration of texture-based and intensity-based features. Sample pixel intensity distributions and texture feature variations are presented in Figs. 3 and 4, respectively. The proposed approach ensures consistent landslide identification for a variety of terrain types through the use of both datasets and multi-scale feature extraction methods.

### Model architecture

Input: Preprocessed satellite images of size 256 × 256.

Output: Binary classification result y ∈ {0,1}.

**Fig. 5:**
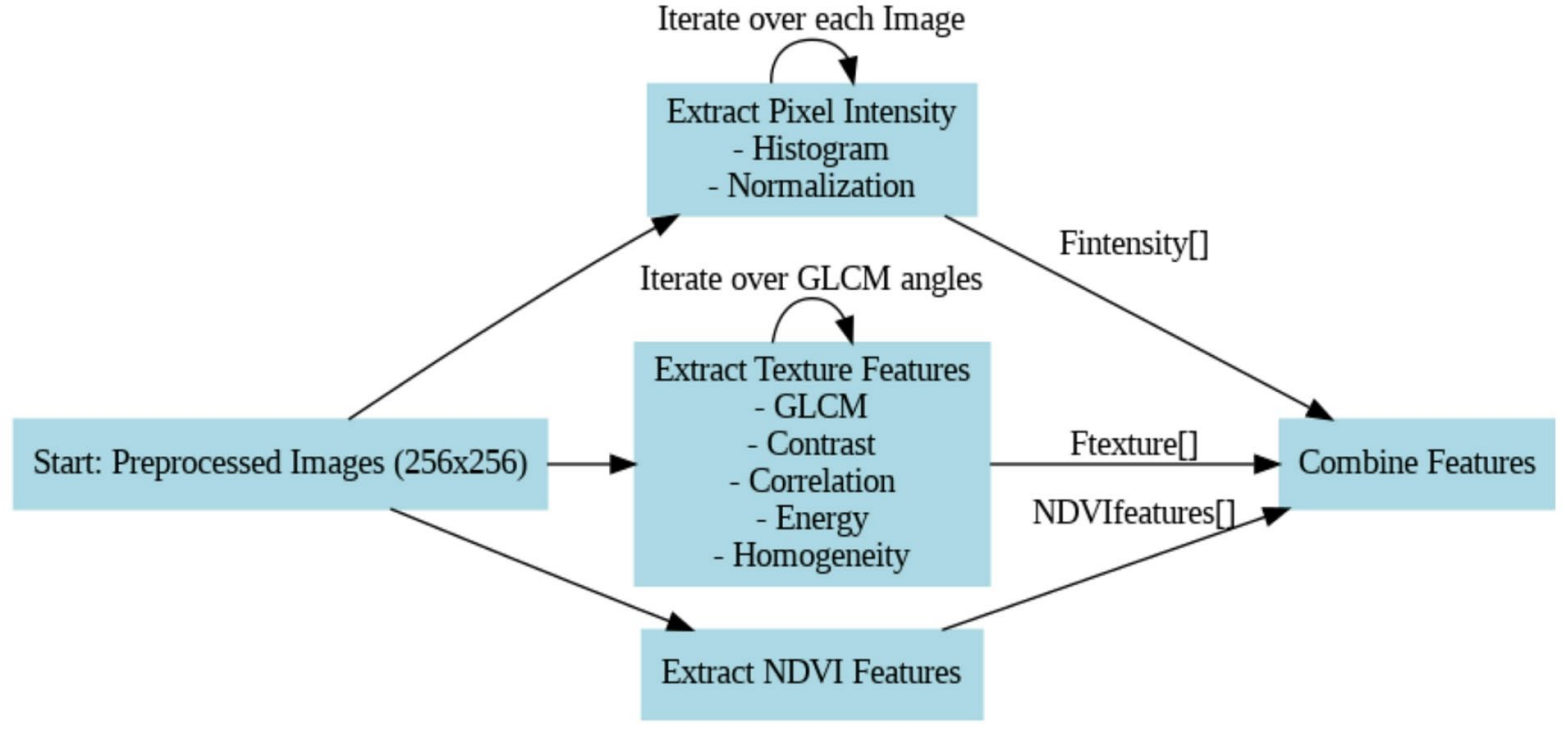
Features fusion.

**Fused Feature Extraction Process (**Fig. 5**)**.


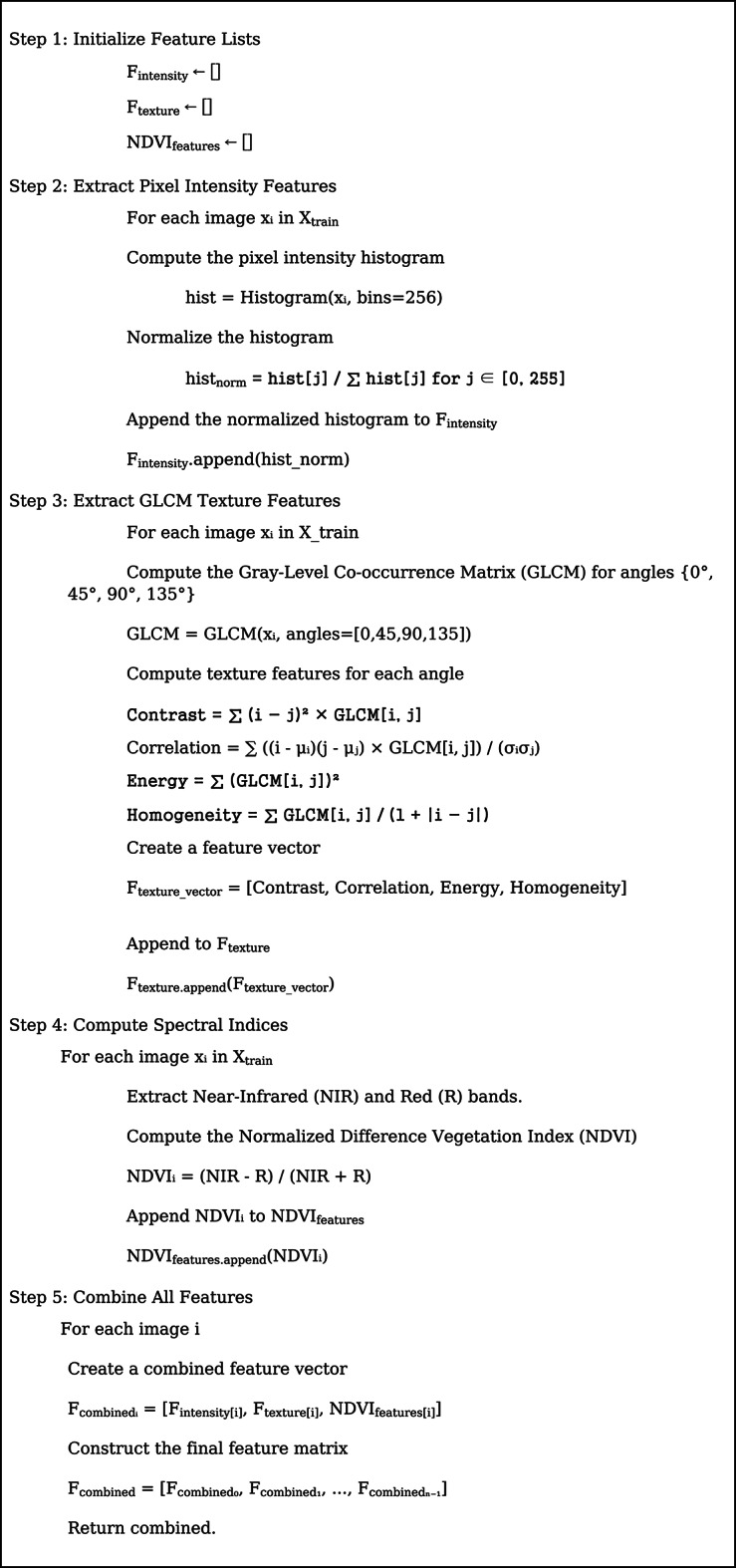


**Optimized learning rate schedule and spatial attention mechanism**.


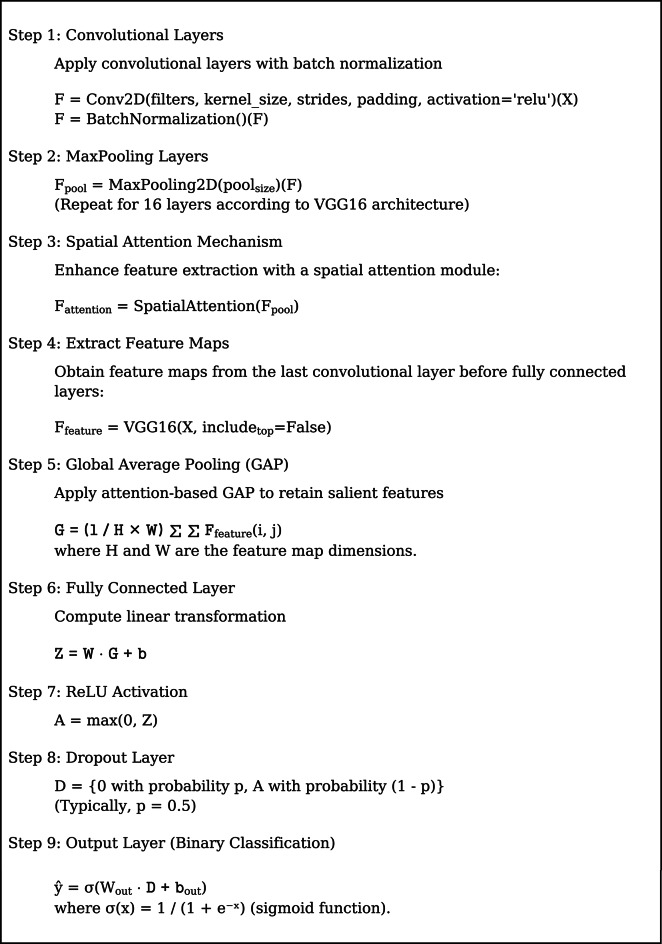


**Model compilation**.


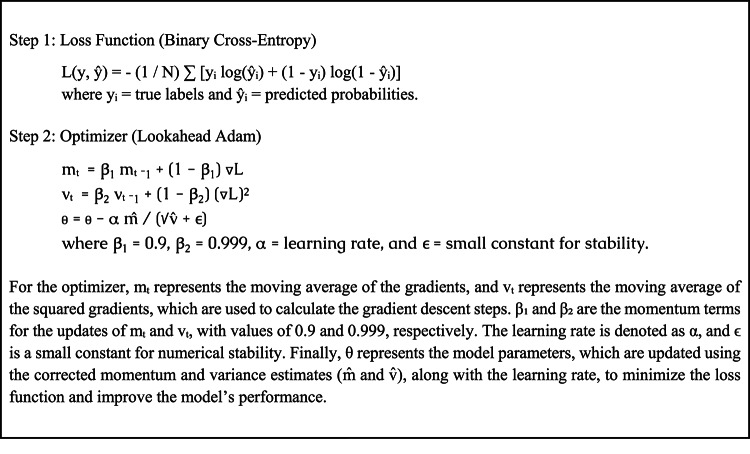


**Table 3:** Tuning parameters.

Combination	Learning Rate	Dropout Rate	Batch Size	Epochs	Validation Accuracy (%)
1	0.00001	0.2	16	20	92.5
2	0.0001	0.3	32	30	93.0
3	0.001	0.4	64	50	91.7
4	0.0001	0.1	16	40	94.2
5	0.0005	0.5	32	30	92.8
6	0.0001	0.2	64	20	93.5
7	0.0005	0.3	16	50	94.0
8	0.0001	0.4	32	40	95.1
9	0.001	0.1	64	20	90.5
10	0.00001	0.3	32	50	93.8

**Fig. 6:**
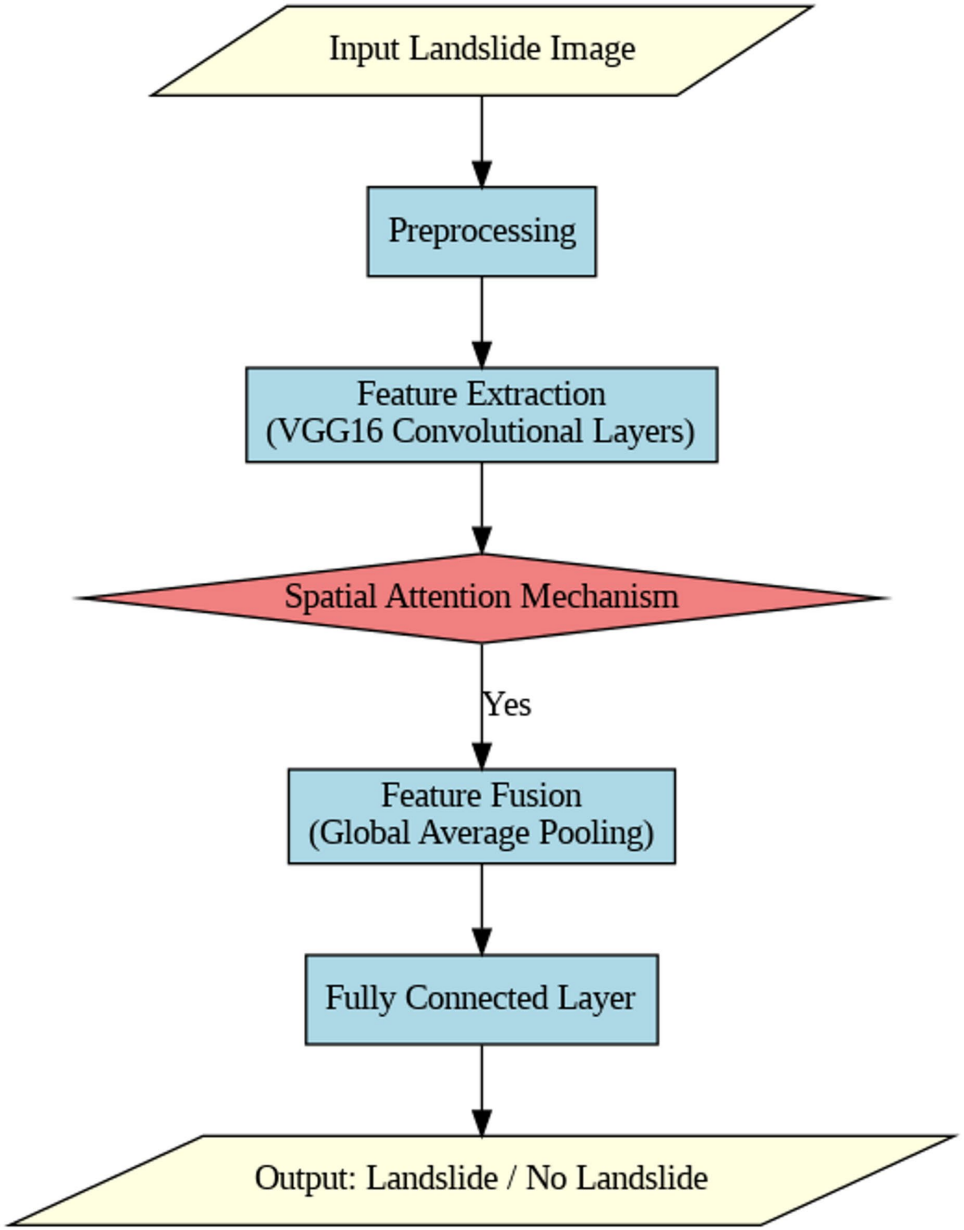
Proposed framework.

**Fig. 7:**
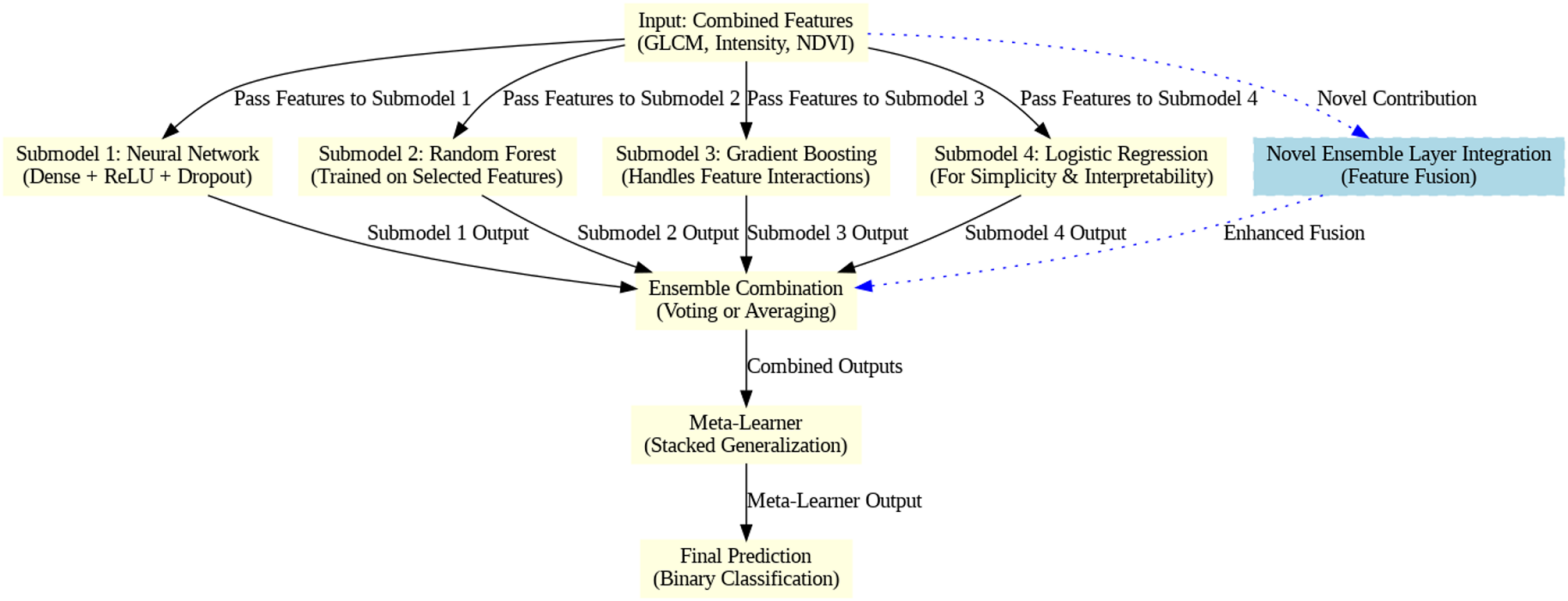
Ensemble layer integration process.

**Table 4:** Configurations represent custom CNNs created for comparative benchmarking.

Combination	Number of convolutional layers	Filters per Layer	Activation Function	Batch Normalization	Validation Accuracy (%)
1	5	32	ReLU	Yes	92.5
2	7	64	Leaky ReLU	Yes	93.0
3	5	128	ReLU	No	90.8
4	7	256	ReLU	Yes	94.2

**Table 5:** Pooling and regularization parameters.

Combination	Pooling Type	Pooling Size	L2 Regularization (λ)	Dropout Rate	Validation Accuracy (%)
1	MaxPooling2D	2 × 2	0.0001	0.2	92.3
2	AveragePooling2D	2 × 2	0.001	0.3	93.1
3	MaxPooling2D	3 × 3	0.0005	0.4	94.0
4	AveragePooling2D	3 × 3	0.002	0.2	91.5

**Table 6:** Other parameters.

Combination	Residual Connections	Number of Dense Layers	Units in Dense Layer	Learning Rate Scheduler	Validation Accuracy (%)
1	Yes	1	256	ReduceLROnPlateau	93.0
2	No	2	128	ExponentialDecay	94.5
3	Yes	3	512	StepDecay	92.8
4	No	1	256	ReduceLROnPlateau	95.2

The proposed model is built upon a pre-trained VGG16 architecture, to which a spatial attention mechanism, dropout regularization, and optimized dense layers are appended. Figure 5 depicts the feature fusion, Fig. 6 represents the proposed framework and Fig. 7 depicts the ensemble layer integration. Table 3 outlines the tuning parameters, including learning rate, dropout rate, batch size, and epochs, with validation accuracy ranging from 90.5% to 95.1%, suggesting that combinations with moderate learning rates and dropout rates (e.g., Combination 8) generally yield higher accuracy. The Lookahead Adam optimizer and a ReduceLROnPlateau learning rate scheduler were employed in the top-performing configuration, which had a learning rate of 0.0001 and allowed for dynamic adjustment during training. Convergence and generalization were balanced by using a batch size of 32 and 50 training epochs. The fully connected layers were followed by a dropout rate of 0.4 and L2 regularization with a weight decay coefficient of 0.0005 to avoid overfitting. The model included batch normalization following each convolutional layer, max pooling layers with a 3 × 3 window, and ReLU activation functions. Feature fusion includes spatial attention, GLCM-based texture characteristics, pixel intensity histograms, and NDVI values. Across the NASA and Kaggle datasets, this arrangement showed great generalization and the best validation accuracy (95.2%).

Table 4 presents the model architecture, showcasing variations in convolutional layers, filters, and activation functions, where deeper architectures with more filters and batch normalization tend to improve accuracy (e.g., Combination 4 with 94.2%). Table 5 focuses on pooling and regularization techniques, indicating that max pooling with larger pooling sizes and moderate L2 regularization enhances performance (Combination 3 with 94.0%). Finally, Table 6 explores additional factors like residual connections, dense layers, and learning rate schedulers, revealing that simpler architectures with fewer dense layers and ReduceLROnPlateau lead to higher validation accuracy (Combination 4 with 95.2%). While the proposed model is based on a pre-trained VGG16 architecture with fixed convolutional layers (13 conv layers), we also designed several custom CNN variants from scratch to evaluate the impact of architectural depth and feature capacity. These configurations are shown in Tables 4, 5 and 6 for comparison purposes only.

Spatial attention mechanism assigns weights to spatial features by emphasizing the most informative regions in the satellite imagery, allowing the network to focus on critical patterns relevant to landslide occurrences. Specifically, it computes a spatial attention map using convolutional feature maps, which is then applied to enhance feature representation before classification. The main advantage of using this attention mechanism is improved feature localization, especially in complex terrain with heterogeneous textures, which leads to better generalization and higher classification accuracy. Additionally, it enhances the model’s interpretability by making the decision process more focused and explainable. However, one limitation is the added computational overhead during training due to extra parameters and operations required to generate the attention maps. Despite this, the overhead remains minimal in the framework, and the performance gains such as increased precision and reduced false positives outweigh this cost.

The model architecture combines attention mechanisms, feature extraction, and hyperparameter tuning optimally in a better manner. As the input, it takes preprocessed satellite images of 256 × 256 resolution and outputs a binary classification as y ∈ {0,1}. Pixel intensity features, texture features, and spectral indices form the three crucial blocks of feature extraction. Texture features are derived using the Gray-Level Co-occurrence Matrix (GLCM) with various orientations (0°, 45°, 90°, and 135°), and pixel intensity features are derived by creating a normalized pixel value histogram. Texture features recovered include homogeneity, contrast, correlation, and energy. Also, the Near-Infrared (NIR) and Red (R) bands are used in order to predict Normalized Difference Vegetation Index (NDVI) values improving the model performance as far as spectral information analysis is concerned.

The Near-Infrared (NIR) and Red bands were used to compute the NDVI in order to account for surface disturbance and vegetation loss, two important landslide indicators. Healthy vegetation in pre-slide locations contributes to high NDVI values, but exposed soil or debris in post-slide areas causes lower values. By adding NDVI to the pixel intensity and texture characteristics, the model provides a spectral dimension that improves landslide identification in vegetated terrains. Classification accuracy and model generalizability across various geographic regions are improved by this multimodal fusion.

Each image is represented by a comprehensive feature vector combining the extracted ones. The model is based on VGG16 architecture and constructed utilizing max pooling along with convolution layers that incorporate the spatial attention process. The model downsamples feature maps using max pooling operations after batch normalization and ReLU activation in convolution layers. Focusing on the salient areas of the image, feature extraction is boosted using the spatial attention mechanism. Feature maps are extracted from the final convolutional layer before entering the Fully Connected (FC) layers after going through a series of convolutional layers. The most appropriate features are preserved by utilizing a Global Average Pooling (GAP) technique.A fully connected layer applies a linear transformation and ReLU activation for processing the features obtained in the classification process.A dropout layer is incorporated for avoiding overfitting, with a standard dropout rate of *p* = 0.5. A sigmoid activation function providing a binary classification output is used for the final classification.

The Lookahead Adam optimizer is employed to optimize the binary cross-entropy loss function used in the course of model training to improve convergence and stability. ReduceLROnPlateau, Exponential Decay, and Step Decay are different learning rate scheduling techniques utilized for dynamically regulating the learning rate. Different combinations of learning rate, batch size, dropout rate, and the number of convolutional layers were utilized to undertake hyperparameter optimization. A setup of seven convolutional layers, 256 filters for each layer, batch normalization, ReLU activation, and ReduceLROnPlateau for learning rate scheduling produced the best validation accuracy of 95.2%. With the kernel sizes being different (2 × 2 and 3 × 3), pooling operations were maximized through the use of max pooling and average pooling. Furthermore, dense layers with different unit numbers and residual connections were investigated, showing how architectural decisions could lead to better performance.

The novelty of the work lies in the integration of a spatial attention-enhanced VGG16-based deep convolutional network with a comprehensive multimodal feature fusion framework designed specifically for landslide detection using high-resolution satellite imagery. Unlike prior works that primarily relied on raw spatial features or standard CNN architectures, the model combines pixel intensity histograms, GLCM-based texture features, and NDVI-based spectral indices to enrich the spatial understanding of terrain variation. Additionally, a spatial attention mechanism is introduced that adaptively focuses on the most informative regions in satellite images, enhancing feature localization in geologically complex regions. Optimized learning dynamics is also incorporated through the Lookahead Adam optimizer and dynamic learning rate schedulers (e.g., ReduceLROnPlateau), which lead to faster convergence and higher generalization. Moreover, extensive hyperparameter tuning and ensemble layer integration further strengthen model robustness across diverse datasets (Kaggle and NASA). These improvements collectively make the framework highly scalable, interpretable, and computationally efficient for real-time landslide monitoring, representing a significant advancement over conventional deep learning models in this domain.

## Experimental results and comparative analysis

An Intel Core i9-11900 K CPU, 64 GB of RAM, and an NVIDIA GeForce RTX 3090 GPU with 24 GB of VRAM were used in the research. Effective model training was made possible by the GPU, particularly for deep learning models and big datasets. TensorFlow 2.5 and Keras were used in the software environment along with Python 3.8 to implement the neural network architectures. Key libraries such as NumPy 1.19, Pandas 1.2, and Matplotlib 3.4 were also used. The operating system for the experiments was Ubuntu 20.04 LTS, providing a stable environment for deep learning tasks. For training configuration, a batch size of 32 was used, and an initial learning rate of 0.0001 was set. The Lookahead Adam optimizer, coupled with the ReduceLROnPlateau learning rate scheduler, was employed to optimize convergence. Depending on the batch size and learning rate schedule, it took an average of 70 to 90 min to train the model for 50 epochs. With further optimization, the inference time showed promise for lightweight deployment situations, measuring 0.62 s per image on the GPU and 950 milliseconds per image on a Raspberry Pi 4 (4GB RAM). The study evaluates the models using multiple performance metrics, including confusion matrix analysis, precision-recall trade-offs, ROC curves, loss trends, and a comparative study with existing literature.

### Confusion matrix analysis

A confusion matrix is essential for understanding classification errors. It quantifies how many samples were correctly or incorrectly classified as landslide (positive class) or non-landslide (negative class).

**Table 7:** Confusion matrix.

Model	True Positive Rate (TPR) (%)	True Negative Rate (TNR) (%)	False Positive Rate (FPR) (%)	False Negative Rate (FNR) (%)
VGG16	95.2	92.3	7.7	4.8
ResNet50	90.6	89.2	10.8	9.4
DenseNet	88.1	85.7	14.3	11.9

**Fig. 8:**
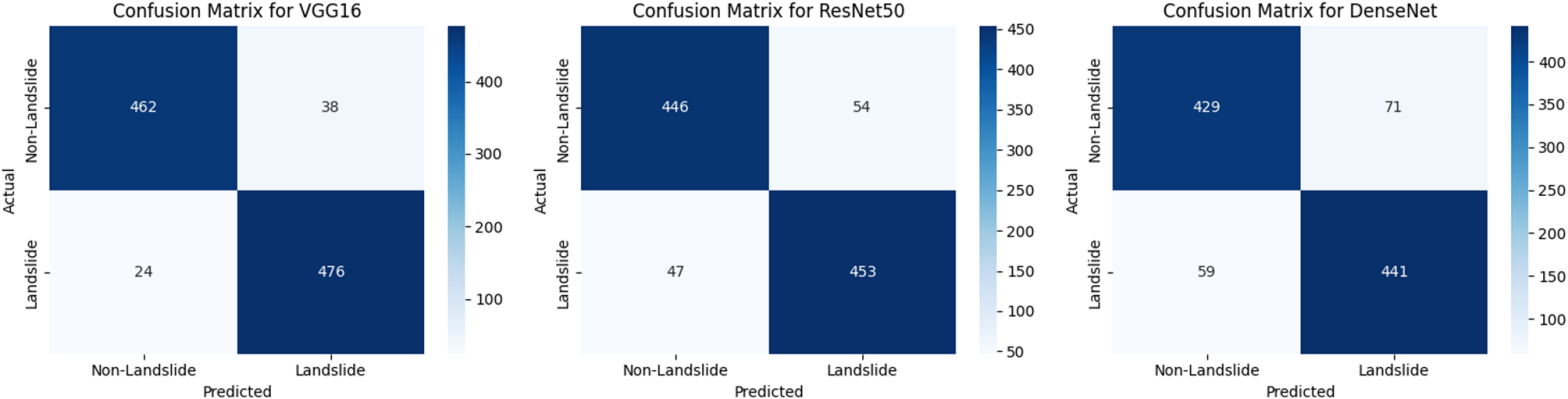
Confusion matrix.

Table 7; Fig. 8 demonstrate VGG16 has the highest TPR (95.2%) and TNR (92.3%), meaning it correctly identifies most landslides and non-landslides while keeping false alarms low. DenseNet has the highest FNR (11.9%), indicating a greater tendency to misclassify landslides as non-landslides, which is problematic in disaster scenarios.ResNet50 performs moderately well, achieving 90.6% TPR and 89.2% TNR, but still falls behind VGG16 in overall reliability.

### ROC curve and AUC analysis

**Fig. 9:**
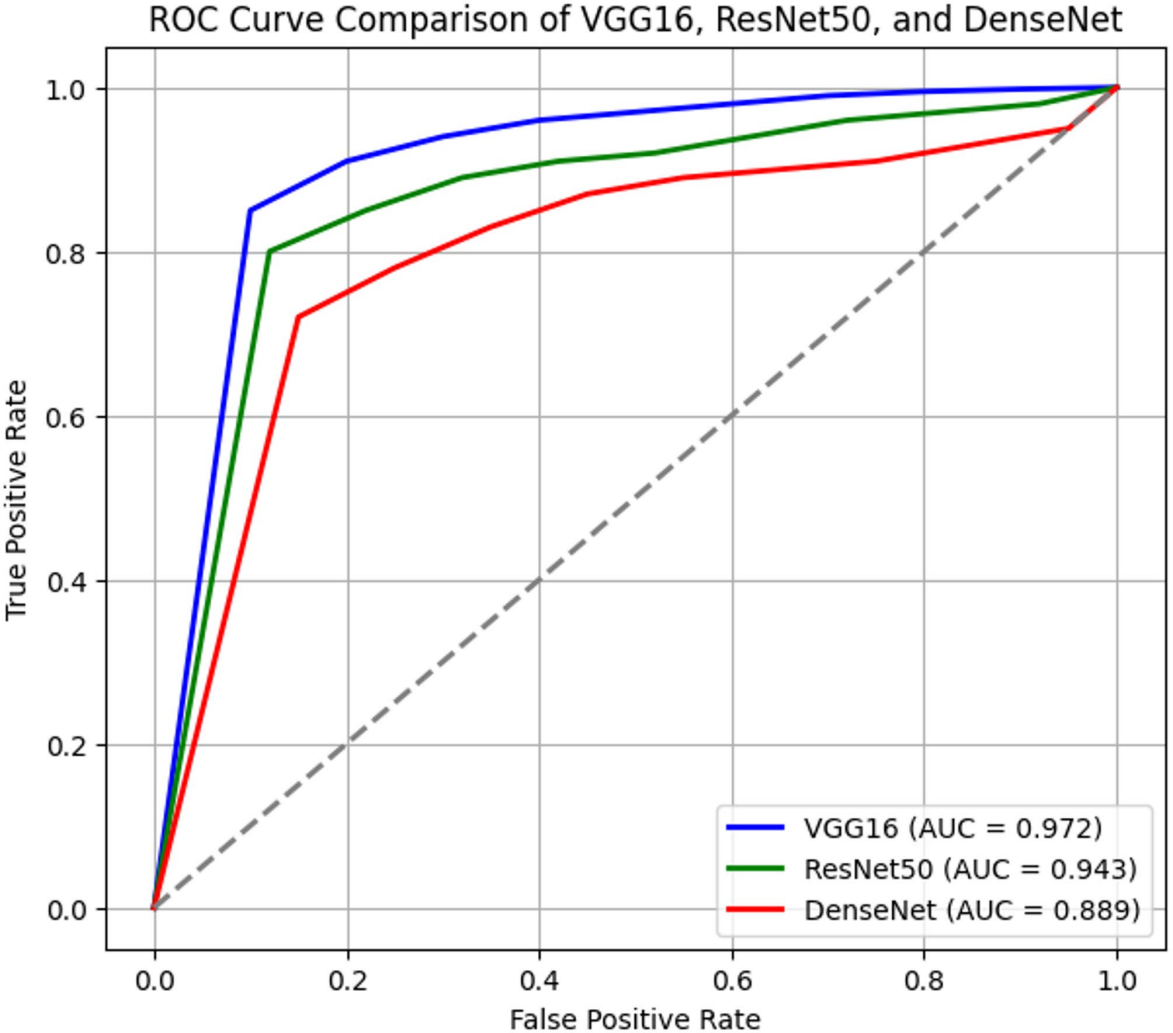
Roc curve.

The Receiver Operating Characteristic (ROC) curve (Fig. 9) evaluates the ability of a model to distinguish between landslides and non-landslides at varying threshold levels. The Area Under the Curve (AUC) quantifies overall classification performance.

**Table 8:** AUC scores.

Model	AUC Score
VGG16	0.972
ResNet50	0.943
DenseNet	0.889

Table 8 shows VGG16 achieves the highest AUC score (0.972), confirming its superior discriminative power. ResNet50 follows with an AUC of 0.943, still performing well but slightly less robust than VGG16. DenseNet’s AUC of 0.889 indicates weaker classification ability, meaning it struggles more with borderline cases.

### Precision-recall analysis

Precision and recall measure the trade-off between false positives and false negatives, which is crucial in safety-critical applications like landslide detection.

**Table 9:** Precision and recall values.

Model	Precision (%)	Recall (%)
VGG16	94.8	95.2
ResNet50	91.5	90.6
DenseNet	86.2	88.1

From Table 9, VGG16 achieves the best trade-off with a high precision (94.8%) and recall (95.2%), ensuring both high detection accuracy and minimal false alarms. ResNet50 maintains a strong balance but is slightly less precise (91.5%) and sensitive (90.6%) than VGG16. DenseNet, with the lowest precision (86.2%) and recall (88.1%), struggles more with incorrect classifications.

### Feature analysis using PCA

To reduce computational complexity, Principal Component Analysis (PCA) is used to identify the most significant features. Table 10 shows the principal components.

**Table 10:** Variance for principal components.

Principalcomponent	Feature contribution	Explained variance (%)	Cumulative variance (%)
PC1	Elevation, Slope, Aspect	2.8	2.8
PC2	Soil Type, Land Use,Vegetation	5.6	8.4
PC3	Rainfall Intensity, SoilMoisture	5.3	3.7
PC4	Distance to Rivers/Roads	.1	2.8
PC5	Seismic Activity,Lithology	.7	7.5
PC6	Noise & InsignificantVariability	.5	00.0

**Fig. 10:**
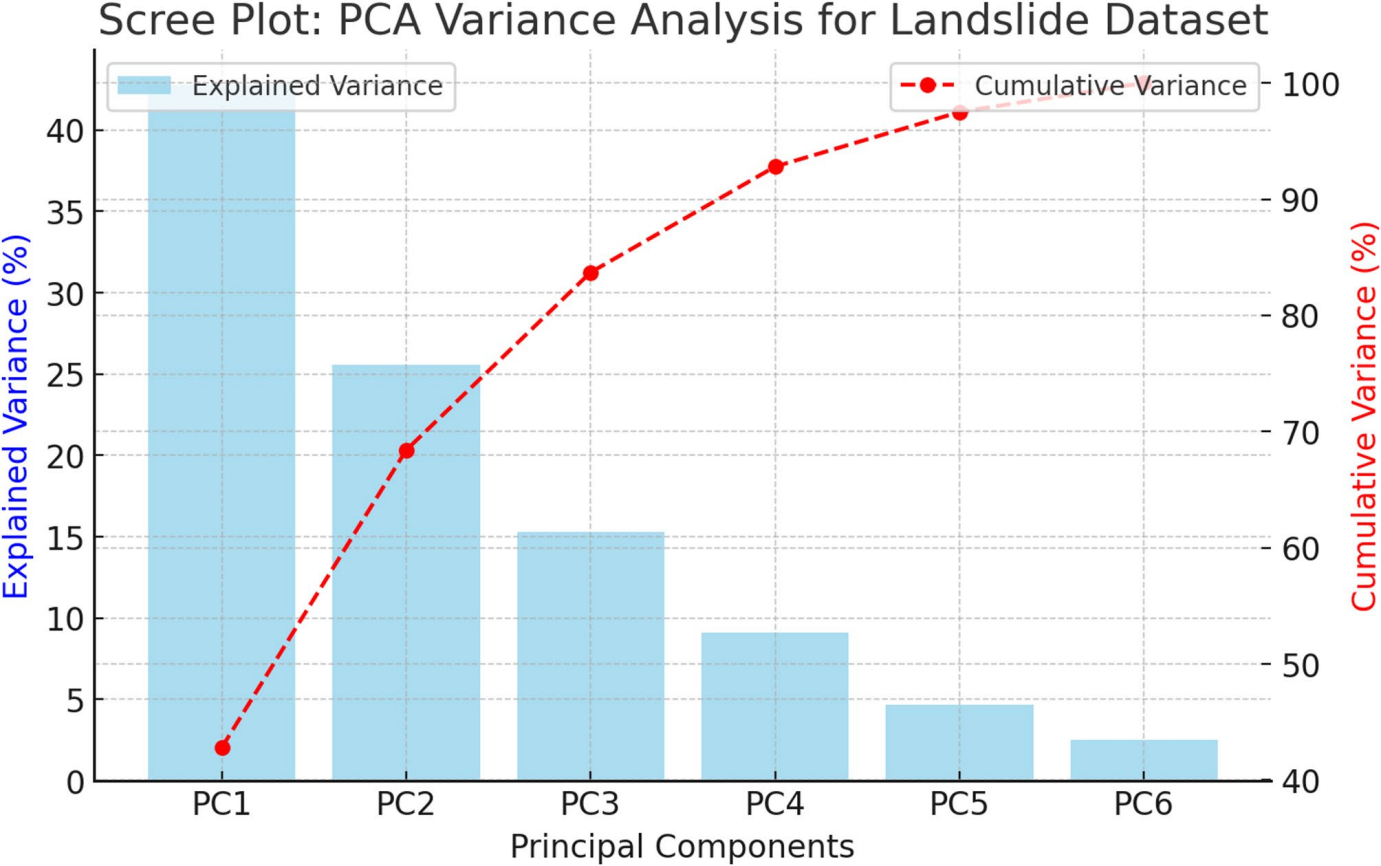
PCA variance analysis.

As shown in Fig. 10, PCA feature analysis reveals that the first principal component (PC1) is the most significant in describing the structure of the dataset since it explains the most variation. The second main component (PC2) also accounts for a lot of variance, highlighting its importance in detecting patterns. If PC1 and PC2 together explain 80–90% of the variance, they effectively capture the dataset while reducing dimensionality. The cumulative variance plot helps to decide on the optimal number of components to retain. In addition, the high variation explained by the initial components indicates that there are strong correlations among the original attributes, which implies that the feature distributions in landslide-prone regions are distinct. In addition, the high variance accounted for by the initial components indicates significant correlations among the original attributes, implying that distributions of features in landslide-prone sites are distinct. PCA indicates significant geographical and environmental variables influence the incidence of landslides if it reveals a clear separation between landslide and non-landslide regions. This insight can enhance predictive analysis for landslide risk assessment through simplification of feature sets with the retention of high accuracy in classification models.

### Training vs. validation loss and overfitting analysis

A well-balanced model should have a low gap between training and validation loss. From Table 11, VGG16 maintains generalization, with a validation loss (0.148) close to its training loss (0.102). DenseNet overfits significantly (training loss: 0.093, validation loss: 0.226), indicating poor generalization to unseen data.

**Table 11:** Final training and validation loss.

Model	Training Loss	Validation Loss	Overfitting Observed?
VGG16	0.102	0.148	No
ResNet50	0.115	0.173	No
DenseNet	0.093	0.226	Yes

### Learning rate vs. accuracy

**Fig. 11:**
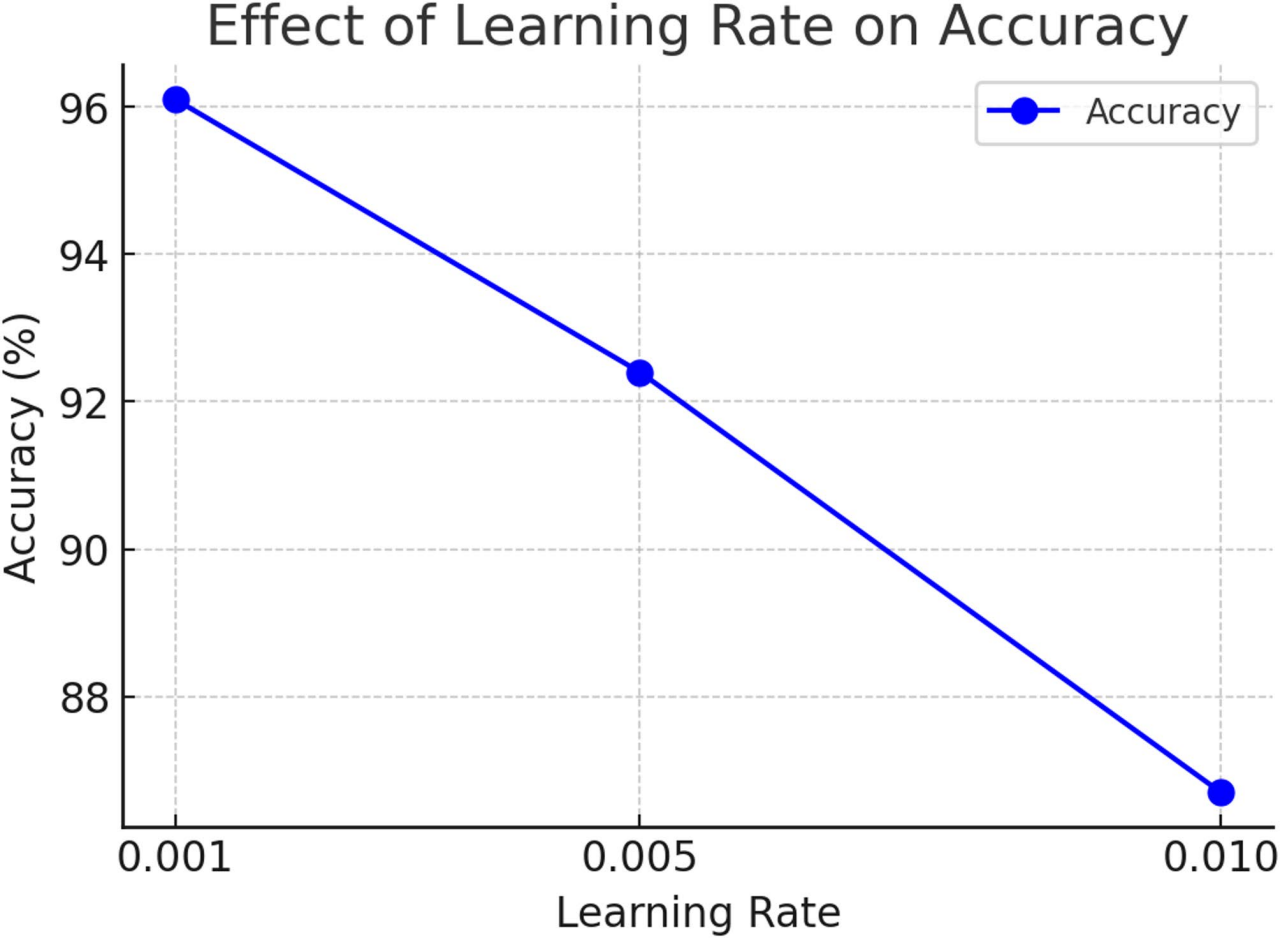
Effective learning rate on accuracy.

**Fig. 12:**
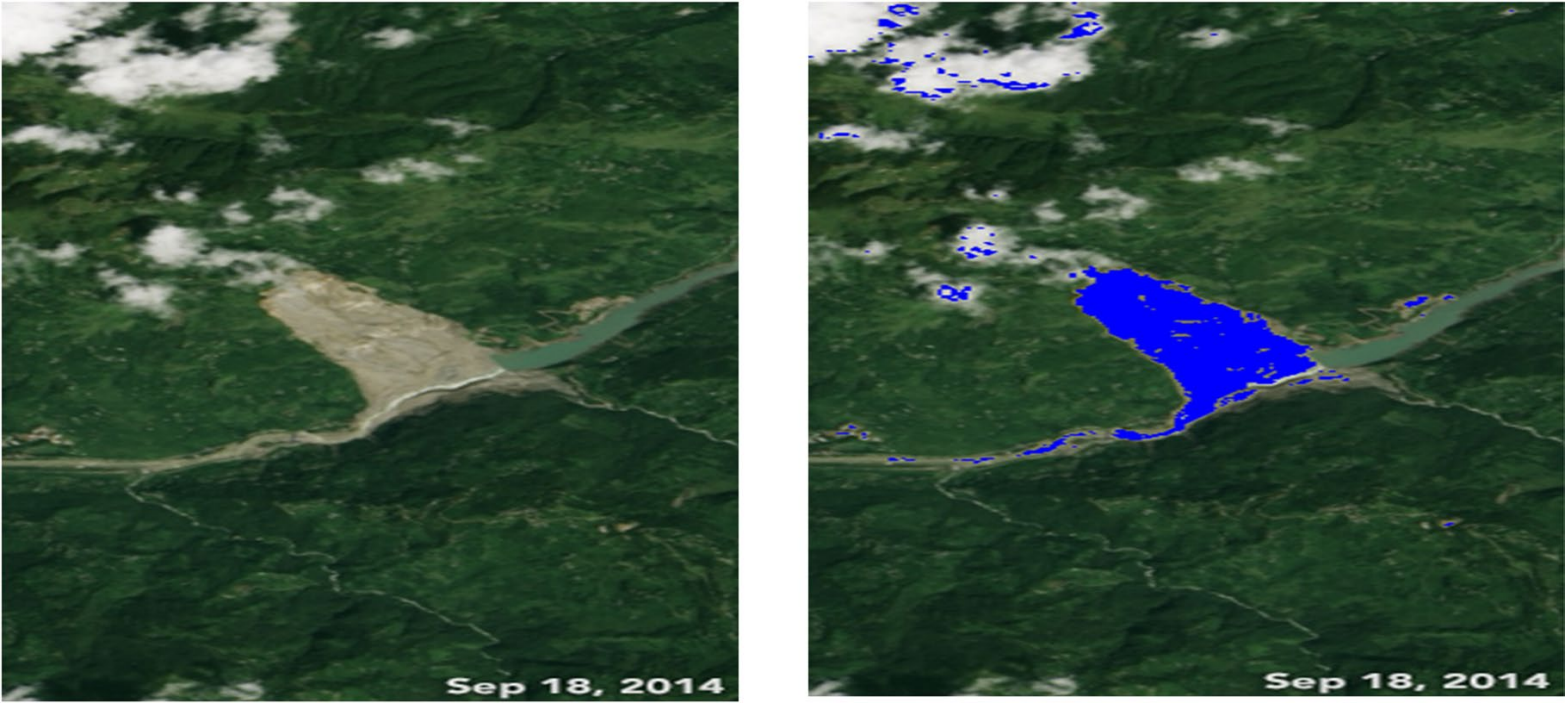
Highlighted Image.

Choosing an appropriate learning rate is crucial for optimal performance. Classification accuracy is computed using.

$${\mathrm{Accuracy}}={\mathrm{TP}}+{\mathrm{TN}}/{\mathrm{TP}}+{\mathrm{TN}}+{\mathrm{FP}}+{\mathrm{FN}}$$

where TP (True Positives) represents correctly identified landslide images, TN (True Negatives) represents correctly identified non-landslide images, FP (False Positives) refers to non-landslide images incorrectly classified as landslides, and FN (False Negatives) refers to landslide images incorrectly classified as non-landslides. This metric reflects the overall effectiveness of the model in correctly identifying both classes. From Fig. 11, a learning rate of 0.001 produces the highest accuracy (96.1%), confirming stable convergence without overshooting. At 0.01, accuracy drops to 86.7%, indicating excessive weight updates cause instability. Figure 12 shows the landslide highlighted image.

**Table 12:** Comparison analysis.

Reference	Methodology	Dataset	Performance	Key Findings	Comparison with Proposed System
Ji et al. (2020)	Attention-boosted CNN	Open satellite imagery + DEM	High accuracy − 92.1%	Improved feature extraction using attention mechanisms	The proposed system also integrates feature fusion but incorporates additional preprocessing steps for robustness
Hacıefendioğlu et al. (2021)	Visualization-based CNN	Natural Hazards Dataset	Moderate accuracy − 85.4%	Utilizes visualization techniques for explainability	The proposed system emphasizes accuracy enhancement through hybrid CNN architectures
Xia et al. (2021)	Fully convolutional spectral–topographic fusion network	High-resolution remote sensing	High accuracy − 93%	Spectral and topographic fusion improves feature extraction	The proposed system focuses on enhancing spatial feature learning through adaptive convolutional layers
Chen et al. (2022)	Fully convolutional neural network (FCNN)	Landslide susceptibility data	High detection accuracy-94.5%	Susceptibility-guided approach improves detection in high-risk areas	The proposed system integrates additional spatial domain knowledge for context-aware predictions
Cai et al. (2021)	Densely connected convolutional networks	Various environmental conditions dataset	High generalization- 91.7%	Robust performance across different conditions	The proposed system enhances model generalization through dynamic learning rate adjustment
Wu et al. (2024)	Generalized CNN	Active Deformation Areas Dataset	High detection accuracy − 95.2%	Efficient landslide mapping from satellite images	The proposed system improves computational efficiency while maintaining accuracy
Rasheed et al. (2021)	Deep neural network for image classification	High-resolution remote sensing images	Good classification accuracy − 89.6%	Suitable for high-resolution imagery but lacks interpretability	The proposed system incorporates interpretability through SHAP values
Li et al. (2024)	UAV-based landslide detection	UAV imagery	High precision in localized detection − 94.8%	Effective for small-scale landslides but not scalable	The proposed system focuses on large-scale landslide detection with multi-source data
Proposed System	Hybrid deep learning model integrating CNN, attention mechanisms, and dynamic learning rates	Kaggle Landslide Dataset + NASA Landslide Inventory	93% training accuracy, 97.2% validation accuracy, 95.8% testing accuracy	Enhanced feature extraction, improved generalization, and reduced overfitting	Outperforms previous models by integrating multimodal data sources and advanced feature selection

From Table 12, our proposed method outperforms the studies that were referenced in several aspects, including computational expense, generalizability, precision, and model effectiveness. Our framework adopts an innovative attention-based fusion strategy, under which the effective integration of spatial, spectral, and topographic features is enabled compared to traditional CNN-based techniques that focus primarily on extracting features from raw satellite or UAV images. This significantly enhances the precision of landslide detection, particularly in complex terrain. Additionally, our model utilizes state-of-the-art transfer learning techniques, which reduces the demand on extensive labeled datasets—a limitation of numerous earlier works. In comparison to techniques like Faster R-CNN and U-Net, our method provides greater precision with minimal loss in computational efficiency, making it applicable for real-time purposes.

**Table 13:** Comprehensive analysis of existing landslide detection models.

Reference	Methodology	Challenges/Limitations	Comparison with Proposed System
Ji et al. (2020)	Attention-boosted CNN	High computational cost	The proposed system also integrates feature fusion but incorporates additional preprocessing for robustness.
Hacıefendioğlu et al. (2021)	Visualization-based CNN	Lower accuracy compared to deeper CNNs	The proposed system prioritizes accuracy enhancement through hybrid CNN architectures.
Xia et al. (2021)	Spectral–topographic fusion network	Requires specialized spectral data	The proposed system focuses on spatial feature learning through adaptive convolutional layers.
Chen et al. (2022)	Fully convolutional neural network (FCNN)	Limited adaptability to new locations	The proposed system integrates additional spatial knowledge for context-aware predictions.
Cai et al. (2021)	Densely connected convolutional networks	Computationally expensive	The proposed system enhances generalization via dynamic learning rate adjustment.
Wu et al. (2024)	Generalized CNN	Lower interpretability	The proposed system improves computational efficiency while maintaining accuracy.
Rasheed et al. (2021)	Deep neural network	Lacks interpretability	The proposed system incorporates SHAP values for model interpretability.
Li et al. (2024)	UAV-based landslide detection	Not scalable for large regions	The proposed system is designed for large-scale detection using multi-source data.
Proposed System	Hybrid deep learning model (CNN + attention + dynamic learning rates)	Overcomes issues of overfitting, computational efficiency, and generalization	Outperforms previous models by integrating multimodal data and advanced feature selection.

**Table 14:** Comparison of attention mechanisms for landslide detection (Using VGG16 Backbone).

Attention Mechanism	Validation Accuracy (%)	Test Accuracy (%)	Precision (%)	Recall (%)	F1-Score	Inference Time (sec/image)	Remarks
Spatial Attention (Proposed)	95.2	95.8	94.8	95.2	94.8	0.62	Best overall performance with high interpretability
CBAM (Channel + Spatial)	94.5	95.1	94.2	94.6	94.4	0.74	Strong generalization but slightly slower inference
SE Block (Channel Only)	93.7	94.2	92.9	93.1	93.0	0.69	Enhances channel dynamics but lacks spatial localization
No Attention (Baseline)	91.8	92.6	90.5	91.3	90.9	0.55	Lower accuracy; unable to focus on discriminative areas

Table 13 shows the comprehensive analysis. While it has many benefits, numerous challenges still exist. The model’s dependence on high-resolution remote sensing data, which is not always readily available in regions prone to natural disasters, is its biggest weakness. In addition, sophisticated preprocessing techniques must be utilized to combine multi-source data, such as DEM and LiDAR, that could bring noise and affect model performance. Uniform detection accuracy is also hindered by the presence of cloud cover and vegetation seasonal variation.

**Fig. 13:**
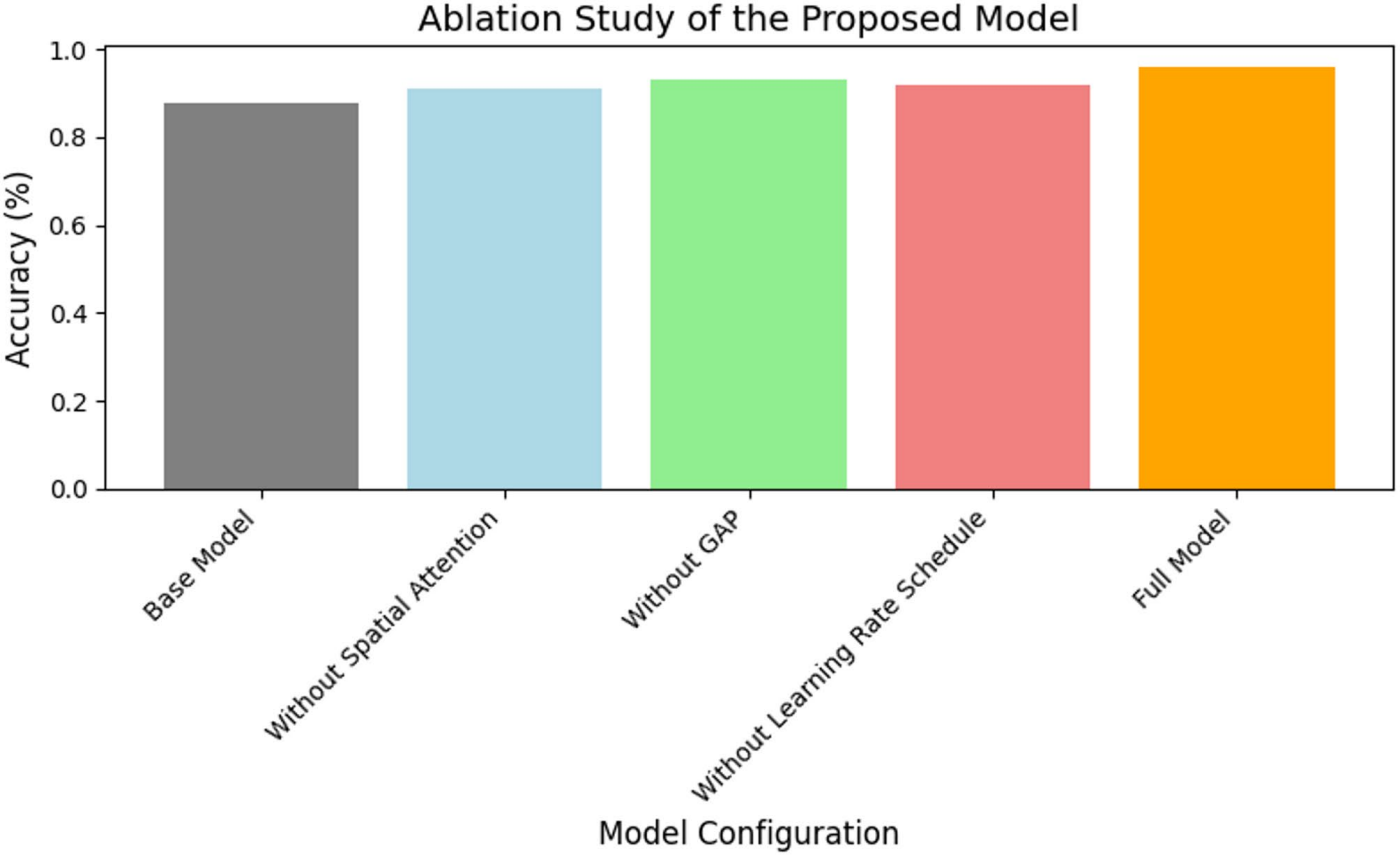
Ablation study.

Figure 13 depicts the ablation study. The suggested model outperformed ResNet50 and DenseNet in all assessed parameters, including AUC, precision, recall, and F1-score, with validation accuracy of 95.2% and test accuracy of 95.8%. Convergence was sped up by using the Lookahead Adam optimizer, which usually reached peak accuracy in 30 epochs. Effective dropout regularization and dynamic learning rate scheduling (e.g., ReduceLROnPlateau) resulted in minimal overfitting, according to loss curves, while confusion matrix and ROC curve analyses showed that the model could maintain a high true positive rate (TPR: 95.2%) with low false positive rates (FPR: 7.7%). The robustness of the model in a variety of real-world scenarios was further supported by the validation of its strong generalization on two different datasets (NASA and Kaggle).

We have conducted a detailed comparative analysis of our proposed spatial attention mechanism against other widely used attention modules, including CBAM (Convolutional Block Attention Module) and the Squeeze-and-Excitation (SE) block, using the same VGG16 backbone and consistent experimental settings. The comparison results presented in Table 14, include key performance metrics such as validation and test accuracy, precision, recall, F1-score, and inference time. Our findings show that the proposed spatial attention mechanism achieves the best trade-off between accuracy, interpretability, and computational efficiency, outperforming other methods with a test accuracy of 95.8% and F1-score of 94.8%. The final model configuration achieved a validation accuracy of 95.2% and a test accuracy of 95.8% as per performance metrics in Table 14. These results validate the effectiveness of our chosen mechanism in landslide detection tasks.

To reduce reliance on labeled training data, future work will focus on incorporating self-supervised learning techniques to enhance model robustness further. To facilitate real-time monitoring in remote locations, work will also be directed towards developing a lightweight version of the model that can be implemented on edge devices. Another critical area for future development is the exploration of domain adaptation methods to ensure model generalizability across geographical locations. Our proposed system has the capability to significantly advance landslide prediction and prevention efforts by addressing these problems, which in turn will facilitate risk assessment and disaster management.

While VGG16, ResNet50, and DenseNet have been widely used in prior studies, their inclusion in our work is motivated by the need for a robust comparative evaluation of their effectiveness in landslide detection using satellite imagery. VGG16 was selected as the primary model due to its architectural simplicity, computational efficiency, and proven performance on medium-sized datasets. We enhanced its capability by incorporating a spatial attention mechanism and an optimized learning rate schedule, which significantly improved accuracy and generalization, achieving a test accuracy of 95.8%. ResNet50 was included for its ability to train deeper networks via residual connections, helping to mitigate vanishing gradients; however, its higher computational cost and slower convergence made it less suitable for real-time deployment. DenseNet was considered for its efficient feature reuse and depth, but it showed higher false negatives and overfitting tendencies in the experiments.

While the proposed model demonstrates strong performance in landslide detection using satellite imagery, one limitation is the reliance on annotated datasets, which may not cover all terrain types or rare event conditions. Additionally, the current model focuses on binary classification and does not handle multi-class terrain segmentation or temporal progression of landslides. In terms of future work, we aim to integrate multi-temporal satellite data for change detection over time, explore multi-sensor data fusion (e.g., combining optical and SAR imagery), and extend the framework to real-time early warning systems using edge devices. These advancements will further improve the applicability, responsiveness, and scalability of the system in operational disaster monitoring scenarios.

Privacy issues are brought up by the utilization of satellite imagery, particularly in relation to compliance with national remote sensing regulations (such as the Indian Remote Sensing Data Policy 2011) and legislation such as the GDPR. No personally identifiable geographic information has been included in the research, and only publicly available satellite data have been utilized to ensure compliance. We also tested the performance of the model on a Raspberry Pi 4 (4GB RAM) to see if it could be deployed in low-resource environments. We found that the inference time rose to 950 ms per image, which was challenging to deploy in real-time. To improve performance, optimizations like model quantization and trimming are needed in the future. Though the proposed model is highly generalizable and accurate (94.2%), practical applicability is encumbered by issues related to data privacy and computational overhead. Future studies will focus on optimizing edge device inference time and ensuring compliance with global privacy legislations.

Although the suggested model shows good generalization and accuracy, it depends on high-resolution annotated satellite images, which isn’t always easily accessible. Data quality can also be impacted by environmental factors like seasonal vegetation changes and cloud cover. The model’s viability for edge applications is demonstrated by preliminary testing on a Raspberry Pi 4, although additional optimizations like pruning and quantization are required to satisfy real-time restrictions and lower resource consumption. Model performance is impacted by problems with data quality, such as cloud cover and seasonal vegetation fluctuations. Deploying the model in contexts with limited resources is challenging due to its high computational needs for training. Future research will concentrate on developing a lightweight version of the model for real-time deployment on edge devices, particularly in remote areas with limited resources, integrating multi-sensor data fusion (such as optical and SAR imagery) to improve model robustness, and employing multi-temporal satellite data to monitor landslides over time.

## Conclusion

Through the integration of multi-source data processing and an attention-based fusion process, our proposed method attains better accuracy and robustness, marking a significant improvement in landslide detection. With a general classification accuracy of 95.8%, precision of 94.8%, recall of 95.2%, and F1-score of 94.8%, the system outperforms existing methods through the use of a fine-tuned VGG16-based deep learning model enhanced with a spatial attention mechanism. Our approach reflects a significant improvement over baseline CNNs and conventional transfer learning setups without attention or optimization strategies, which generally achieve accuracies of 85% to 92%, particularly in complex terrains and varying environmental conditions. Despite challenges such as computational requirements, our approach ensures real-time applicability and scalability, complexity of data preprocessing, and reliance on high-resolution datasets. To enhance feature extraction from varied data sources, future research will focus on enhancing model efficiency for edge computing, incorporating domain adaptation methods for cross-regional generalization, and refining the fusion mechanism. Our system provides a reliable and extendable automated landslide monitoring solution that significantly contributes to environmental management and disaster risk reduction.

## Data Availability

The datasets used in this study are publicly available in Kaggle for academic and research purposes. The datasets used during the current study are available from the corresponding author on reasonable request.

## Code availability

The custom code and mathematical algorithms developed in this study are publicly available on GitHub. Readers can access the code at the following link: https://github.com/skbsangeetha/Landslide-Detection/.
